# Spontaneous Seizure Outcomes in Mice Using an Improved Version of the Pilocarpine Model of Temporal Lobe Epilepsy

**DOI:** 10.3390/ijms26199540

**Published:** 2025-09-29

**Authors:** Ronald P. Gaykema, Madison J. Failor, Aleksandra Maciejczuk, Magda Pikus, Mariia Oliinyk, Maggie B. Ellison, Amir A. Behrooz, Kiran Singh, John M. Williamson, Edward Perez-Reyes

**Affiliations:** 1 Department of Pharmacology, University of Virginia, Charlottesville, VA 22903, USA; 2 Biomedical Sciences Graduate Program, University of Virginia, Charlottesville, VA 22903, USA; 3 Department of Neurology, University of Virginia, Charlottesville, VA 22903, USA; 4 UVA Brain Institute, University of Virginia, Charlottesville, VA 22903, USA

**Keywords:** temporal lobe epilepsy, electroencephalogram (EEG), spontaneous seizures

## Abstract

Temporal lobe epilepsy (TLE) is a debilitating disorder that affects millions of people worldwide and is difficult to treat with medicines. There has been little progress in the development of novel therapies for these patients because of the lack of suitable animal models. Current rodent models of TLE use chemoconvulsants or electrical stimulation to induce status epilepticus, which evolves into chronic epilepsy with spontaneous recurring seizures. These models have face validity in human TLE as they share similarities with seizure onset in the hippocampus, EEG patterns, tonic–clonic convulsions behavior, and hippocampal sclerosis. Unfortunately, seizure frequencies are so variable that they hinder drug testing. The ideal model for screening epilepsy therapies would have spontaneous seizure frequencies that are greater than two per day, little-to-no seizure-free days, and would maintain these features for more than 4 weeks. This study describes a series of improvements to the mouse pilocarpine TLE model. First, a pharmacokinetic model was developed to guide pilocarpine dosing. Second, induction was combined with EEG monitoring, allowing for real-time monitoring of pilocarpine-induced EEG discharges and electrographic seizures that precede behavioral manifestations. Third, strains of mice were identified that withstand pilocarpine-induced status epilepticus and reliably develop spontaneous recurring seizures. The pilocarpine model was improved by lowering mortality and increasing the fraction of mice that developed spontaneous seizures and had seizure frequencies that are amenable to drug screening. Future studies are required to identify the ideal mouse strain for drug screening and validate the response to known anti-epileptic drugs.

## 1. Introduction

Epilepsy is a major neurological disorder with significant economic and human burdens. The prevalence of active epilepsy has been estimated at 1.2%, which translates into 96 million people worldwide [1]. In addition to the economic burden, epilepsy patients have a reduced quality of life [2]. Temporal lobe epilepsy (TLE) is the most common type of focal epilepsy. Seizure onset zones reside in hippocampal and parahippocampal brain regions. The hippocampus is particularly vulnerable to seizures, and human TLE can be caused by a wide variety of insults such as febrile seizures, status epilepticus, dysplasia, infection, stroke, and traumatic brain injury [3,4]. Despite the gravity of this problem, medical treatment of TLE fails in roughly a third of patients due to ineffectiveness, the development of pharmacoresistance, or intolerance to side effects [5,6]. The only option left for some of these patients is surgical resection of the temporal lobe. Despite its effectiveness, there are many drawbacks to this surgery. First, only about a third of these patients are eligible for surgery [7] and there are significant barriers to treatment such as access [8]. Second, surgery is only effective in controlling seizures in two-thirds of patients and, after 5 years, only half remain seizure-free [9,10]. Third, surgery disrupts verbal memory in one-third of patients [11]. Clearly, there is a great need to develop novel anti-epileptic therapies, a conclusion shared by the American Epilepsy Society Basic Science Committee, the International League Against Epilepsy Working Group for Preclinical Epilepsy Drug Discovery, and the National Advisory Neurological Disorders and Stroke Council [12,13].

Numerous animal models of temporal lobe epilepsy have been developed in rodents. These models begin by inducing long-lasting status epilepticus (SE), either by using chemoconvulsants (e.g., kainic acid, pilocarpine) or prolonged electrical stimulation [14]. Evidence that these animal models are valid proxies for human TLE include EEG patterns, tonic–clonic motor seizures, hippocampal sclerosis, and localization of the seizure focus [15]. These models were originally developed in rats. Adapting these models to mice has been difficult because mice often die during pilocarpine-induced SE [16]. This problem is compounded by the requirement for >1 h of SE to develop epilepsy with spontaneous seizures [17]. Animals that survive SE and develop spontaneous recurring seizures (SRSs) show extensive neuronal death throughout the brain [18,19]. Seizure frequency in animal models varies greatly even within the same cohort of animals, and seizures often cluster Two common seizure patterns that hamper drug screening have been observed: clustering and progression. A cluster of seizures can be operationally defined by a sudden increase in the number of seizures per day over baseline, often followed by many seizure-free days [20,21,22]. Progression can be defined by a time-dependent increase in seizure frequency or other measures of seizure severity such as seizure duration or behavioral score. From these studies, we conclude that current models of TLE are inadequate for large-scale drug screening, mainly due to the low frequency of SRSs and seizure clustering with long gaps of seizure-free days. The present study describes a pharmacokinetic model to guide pilocarpine dosing in different strains of mice and characterizes the seizure patterns in chronically epileptic mice.

## 2. Results

### 2.1. Seizure Patterns in Three Models of TLE

**Figure 1:**
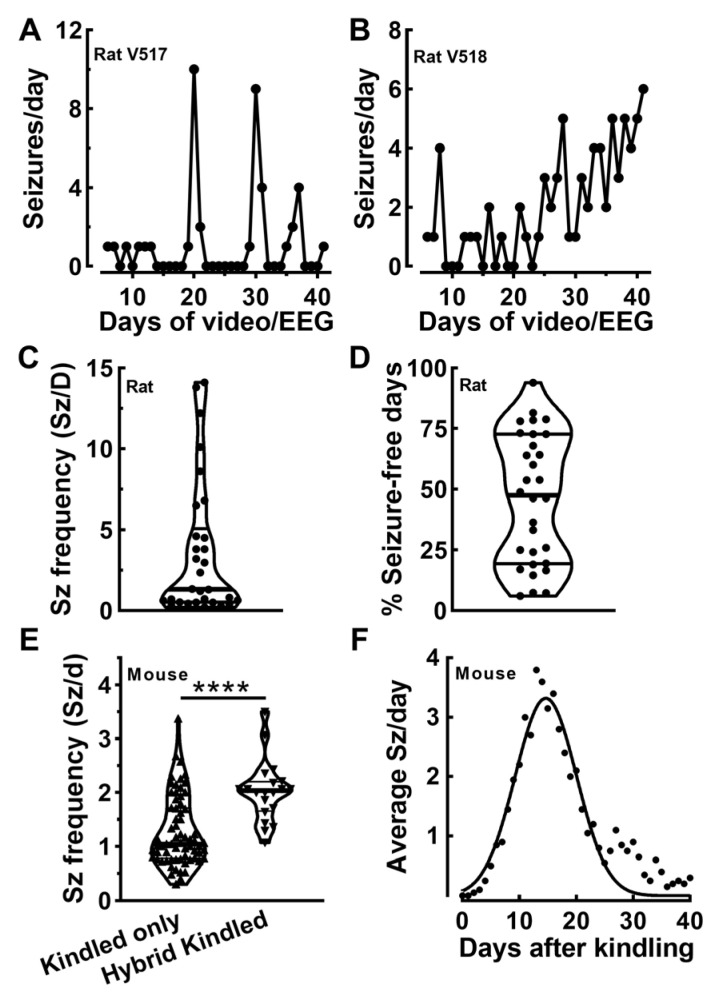
**Spontaneous seizures in the rat pilocarpine model are highly variable, showing clustering and progression.** Seizure diaries from individual rat subjects are shown in panels (**A**) (V917) and (**B**) (V518). (**A**), A representative seizure diary with clusters, which can be defined as days with greater than 7 seizures followed by 5 or more seizure-free days. (**B**), A representative seizure diary with progression, which is defined as an increasing seizure frequency over time. (**C**), The average seizure frequencies observed in several cohorts of pilocarpine-treated rats (*n* = 30). (**D**), The average numbers of days that rats did not have a seizure over 40 days of EEG recording. Seizure outcomes using hybrid kindled VGAT-Cre mice (**E**,**F**). (**E**), Comparison of seizure frequencies in VGAT-Cre mice that were either electrically kindled or hybrid kindled, which combines a single treatment with kainic acid (IP) and electrical kindling. Statistical comparison by Mann-Whitney U test (*p* < 0.0001, *n* = 67, kindled; *n* = 20 hybrid kindled). (**F**), Epilepsy is transient in hybrid kindled VGAT-Cre mice. The line represents Gaussian fit (r^2^ = 0.91). Abbreviations are as follows: ****, *p* < 0.0001.

The goal of our study is to develop novel therapies for patients with temporal lobe epilepsy [23]. Initial studies used the rat Li/pilocarpine model to induce status epilepticus that evolves into chronic epilepsy with spontaneous recurring seizures. Our intention was to screen drug-inducible gene therapies for their anti-seizure properties in chronically epileptic animals. Although these studies successfully showed the anti-seizure potential of AAV-SmonCeiT, which expresses *Kcnk2* leak potassium channels, these studies were not performed on epileptic animals [23]. Attempts to screen epileptic animals failed due to the high variability in seizure frequencies in the rat Li/pilocarpine model (Figure 1). We observed two common patterns of seizures in these studies: (1) seizure clusters with ~10 Sz/d followed by many seizure-free days (Figure 1A); and (2) seizure progression where the number of seizures per day rose throughout the recording period. Both patterns complicated the interpretation of any before-and-after drug treatment comparison. Seizure frequencies in rats treated with the Li/pilocarpine protocol were highly variable (Figure 1C). Similarly, the percentage of days that individual rats did not have a seizure varied from 6 to 94% (Figure 1D). We concluded that the rat Li/pilocarpine model produced spontaneous seizure patterns that were not amenable to drug screening.

We next turned to electrical kindling models with the goal of testing the GABA hypothesis of epilepsy [24]. Our strategy was to express *Kcnk2* in a Cre-dependent manner in hippocampal dentate hilar neurons in VGAT-Cre mice [25]. Unexpectedly, we discovered that VGAT-Cre mice developed spontaneous seizures after electrical kindling, even without *Kcnk2* expression [26]. To improve the seizure frequencies in kindled VGAT-Cre mice (Figure 1E), we developed a hybrid kindling approach that combines kainic acid with electrical stimulation. Using this approach, we were able to double the average seizure frequency. Unfortunately, we discovered that epilepsy in VGAT-Cre mice spontaneously remits (Figure 1F); spontaneous seizures increase in frequency over the first 2 weeks, reach a peak around 3 Sz/d, and then gradually diminish over 4 weeks. Again, we concluded that this seizure pattern is not amenable to drug screening.

### 2.2. Development of Pharmacokinetic Model for Pilocarpine Dosing

**Figure 2:**
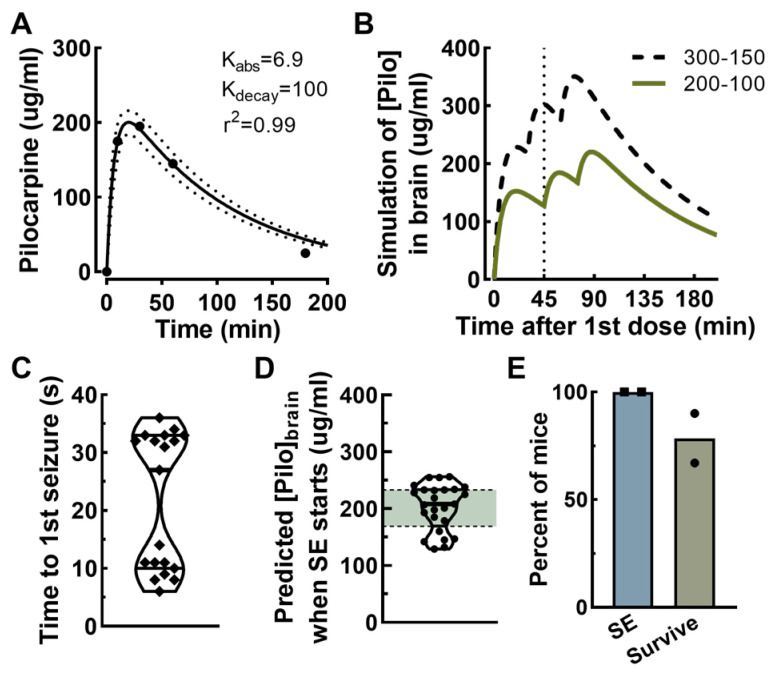
**A pharmacokinetic model of pilocarpine concentrations in the brain to guide dosing in mice.** (**A**) The PK-Pilo model is based on the results from Mazuferri et al., 2012 [30]. Their results were fit by eye to a double exponential equation to estimate the rate of brain penetration (absorbance, abs) and elimination (decay). Dotted lines represent the 95% confidence levels. (**B**) Application of the PK-Pilo model to estimate pilocarpine brain concentrations after repeated dosing. The 300–150 lines are based on the pilocarpine dosing scheme used by Vigier et al., 2021 [27] for Swiss/CD-1 mice. (**C**) A plot of the time to the first seizure after pilocarpine injection in CD-1 mice that did not require a second injection (*n* = 19). (**D**) In CD-1 mice that developed SE, we calculated the brain concentration of pilocarpine at the start of SE (*n* = 25). Green shaded area represents the 25-75% quartiles, which is the target range for brain pilocarpine concentrations. (**E**) The percentages of CD-1 mice that survived pilocarpine treatment and SE. The experiment was performed on 2 cohorts, and the results of each trial are shown by symbols (mean ± sem, *n* = 21 mice).

Preliminary studies using pilocarpine to induce status epilepticus in VGAT-Cre mice resulted in high mortality, a known drawback to this model [16]. Recent advances on the use of pilocarpine in mice to induce SE used an initial high dose followed by lower doses [27,28]. We reasoned that these protocols induced SE by increasing brain concentrations of pilocarpine to the point of inducing SE without causing death, which is due to tonic contraction of the diaphragm leading to fatal apnea [29]. To provide a rational dosing scheme, we developed a pharmacokinetic model of pilocarpine absorption and decay. This “PK-Pilo” model was made possible due to the careful studies of Mazufferi et al. [30] which measured brain concentrations of pilocarpine in mice after flushing the brain vasculature. Figure 2A shows a replot of their measured pilocarpine concentrations in the brain fit to the data using two exponentials, one for absorption (Kabs = 6.9) and the second for decay (Kdecay = 100). We ran various simulations with this model to test the effect of repeated dosing, varying both the dose and intervals between doses (Figure 2B). These dosing schemes were influenced by the results of Vigier et al. [27] using Swiss mice, an outbred strain also called CD 1. We noted that CD-1 mice have also become the strain of choice for post-traumatic epilepsy studies [27,31]. Testing the PK-Pilo model in CD-1 mice yielded encouraging results (Figure 2C–E). An important finding was that the delay between pilocarpine injection and the first seizure appears to be a bimodal distribution with clusters at the 25th and 75th percentile, which corresponds to 10 and 33 min, respectively (Figure 2C). To avoid dosing a mouse that was about to start seizing, we extended the time to the second dose to 45 min (Figure 2B). Figure 2D plots the predicted pilocarpine concentration in the brain at the time when behavioral status epilepticus starts. This analysis defines a range between 170 and 230 μg/mL pilocarpine in the brain where SE is triggered in CD-1 mice. Importantly, the PK-Pilo protocol triggered SE in all mice, with a high survival rate of 75% (Figure 2E).

### 2.3. Pilocarpine Induction with EEG

**Figure 3:**
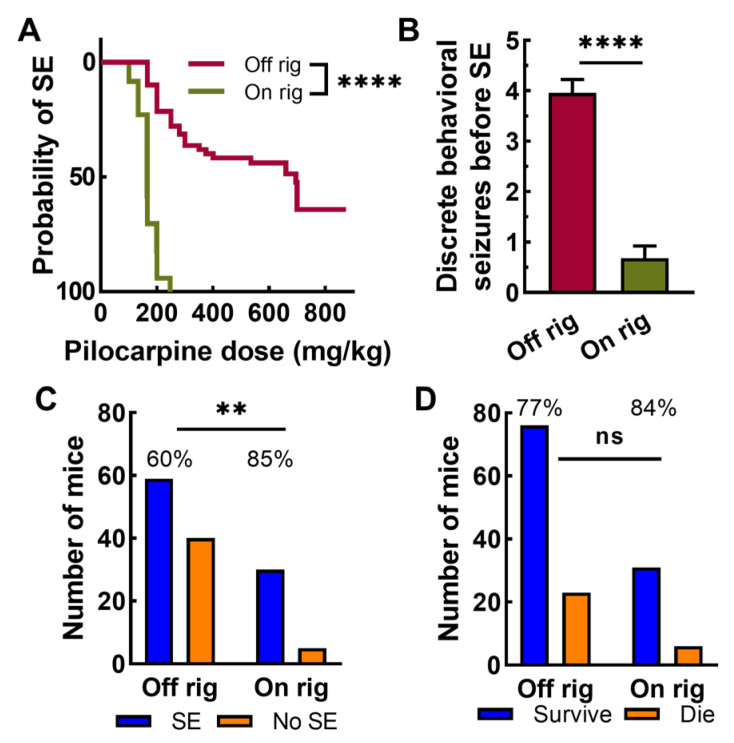
Pilocarpine induction of status epilepticus differs dramatically when mice are implanted with an EEG recording headset and recorded (on-rig). (**A**) A Kaplan–Meier plot showing the pilocarpine dose required to trigger SE. Pilocarpine induction of SE occurs at lower total pilocarpine doses on rig (Mantel–Cox test, *n* = 22 on-rig, *n* = 62 off-rig). (**B**) Pilocarpine injected into mice with no headset (off-rig) showed more discrete seizures before SE onset (Mann–Whitney test). (**C**) Chi square analysis of the mice that either developed SE or did not under the two conditions (Fisher’s exact test). (**D**) Chi square analysis of the mice that either survived pilocarpine treatment or did not under the two conditions (Fisher’s exact test). Neither data set passed the Anderson–Darling test of normality, so statistical analysis used the Mann–Whitney *t*-test. The pooled results from strains used in subsequent experiments. Abbreviations are as follows: ns, not statistically significant; **, *p* < 0.01; ****, *p* < 0.0001.

Emboldened by the high survival rates, we next studied pilocarpine induction of SE in mice implanted with an EEG recording headset and monitored by video/EEG. Surprisingly, mice dosed while on the EEG rig (“on-rig”) required lower pilocarpine doses to trigger SE compared to off-rig mice (Figure 3A). Most on-rig mice went into SE at a dose of 166 mg/kg pilocarpine, while only 10% of off-rig mice went into SE at this dose. A second dramatic difference was that off-rig mice had many more discrete pilocarpine-induced seizures than on-rig mice (Figure 3B). In many cases, on-rig mice went straight into SE. The ability to induce SE on rig was higher compared to off rig, a result that was statistically significant. Survival rates remained high under both conditions, with no statistical difference (Figure 3D).

### 2.4. Strain Dependence of Pilocarpine Induction on Rig

**Figure 4:**
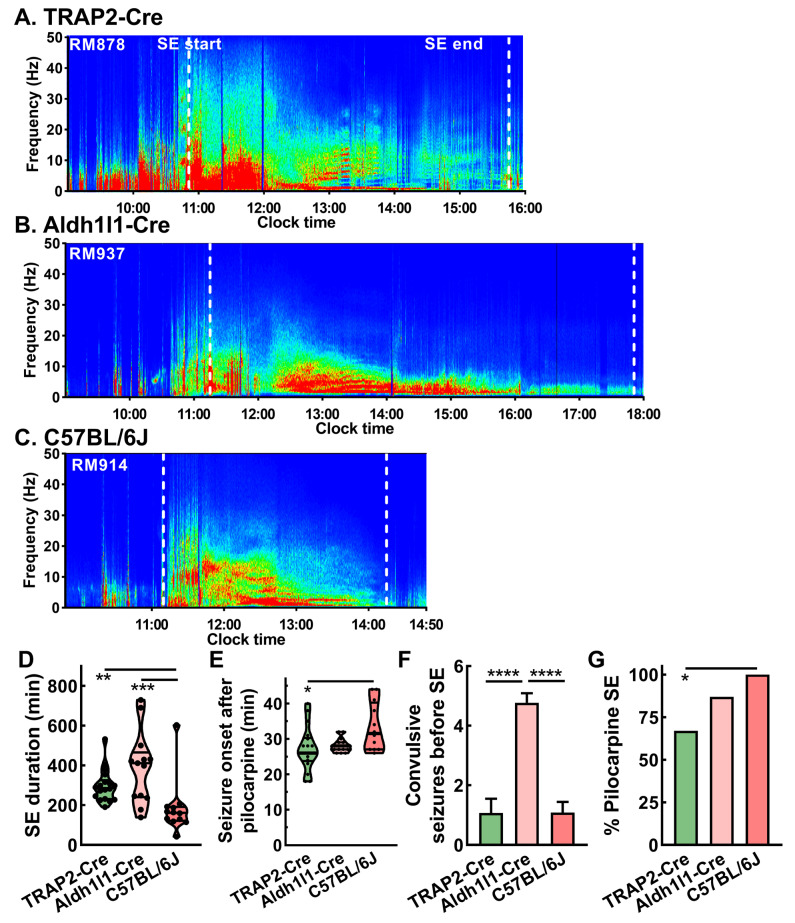
Pilocarpine induction of status epilepticus while on rig for 3 strains of mice. Representative EEG power spectra during pilocarpine induction in the following 3 mouse strains: (**A**) TRAP2-Cre x CD-1, (**B**) Aldh1l1-Cre x CD-1, and (**C**) C57BL/6J. Dotted white lines represent the start and end of status epilepticus. The inset shows the coloring scheme of power spectra. (**D**) The duration of status epilepticus varied significantly, following the rank order Aldh1l1-Cre > TRAP2-Cre > C57BL/6J. (**E**) The time between initial pilocarpine dosing and the 1st seizure was similar across the 3 strains. A small statistical difference was noted between TRAP2-Cre and C57BL/6J mice. Statistical analysis by one-way ANOVA followed by the Kruskal–Wallis test. (**F**) The response to pilocarpine injection begins with a decrease in locomotion, which is then followed by discrete short (~30 s) electrographic seizures with tonic–clonic motor seizures. These seizures are followed by a return to EEG baseline. Aldh1l1-Cre mice had significantly more induced seizures (*n* = 15) than either TRAP2-Cre (*n* = 18) or C57BL/6J (*n* = 12) mice. Statistical analysis by one-way ANOVA followed by the Kruskal–Wallis test. (**G**), The percentage of mice that developed status epilepticus is around 80%, with a small yet statistically significant difference between TRAP2-Cre and C57BL/6J mice. Statistical analysis on strain pairs by Chi square analysis followed by Fisher’s exact test. Abbreviations are as follows: *, *p* < 0.05; **, *p* < 0.01; ***, *p* < 0.001; and ****, *p* < 0.0001.

We next studied the strain dependence of pilocarpine induction of status epilepticus. Representative power spectra illustrate the duration of SE and how it varies between strains (Figure 4A–C). C57BL/6J mice showed the shortest SE duration, while Aldh1l1-Cre mice showed the longest duration of SE. TRAP2-Cre mice showed an intermediate duration of SE that was statistically longer compared to C57BL/6J but not statistically different compared to Aldh1l1-Cre mice (Figure 4D). Seizure onset after pilocarpine administration was approximately 30 min for all three strains, with statistical difference between TRAP2-Cre and C57BL/6J (Figure 4E). Aldh1l1-Cre mice showed more discrete pilocarpine-induced seizures than the other 2 strains (Figure 4F). There was a statistical difference noted between TRAP2-Cre and C57BL/6J in terms of the fraction of animals that went into status epilepticus (Figure 4G).

### 2.5. Strain Dependence of Seizure Patterns in Epileptic Mice

**Figure 5:**
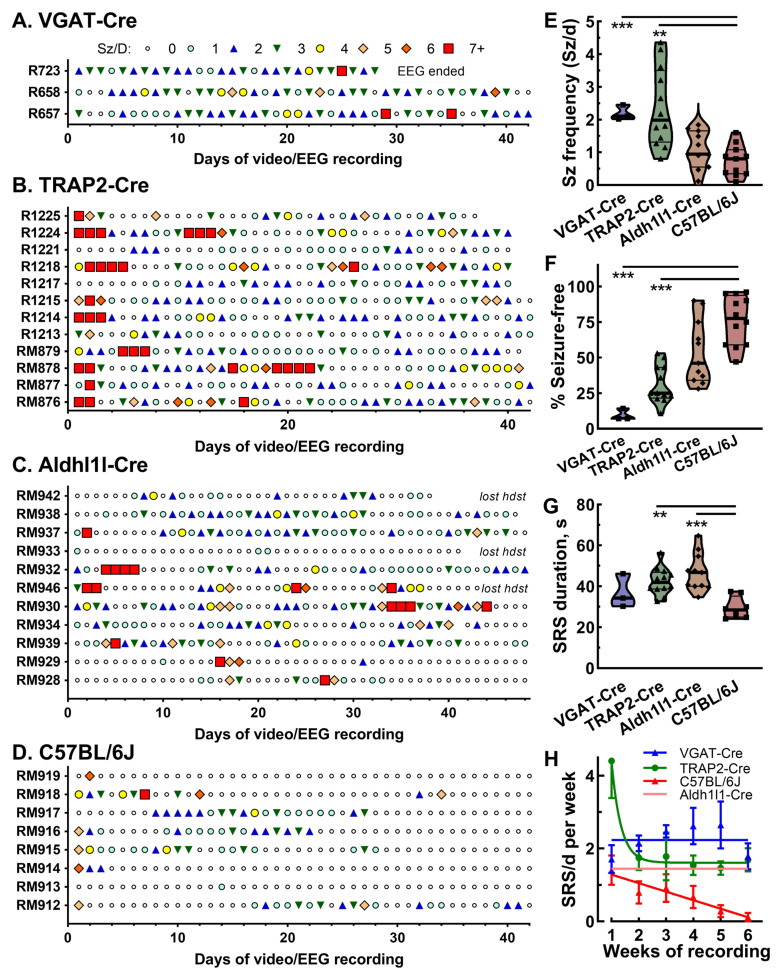
**Spontaneous recurring seizure outcomes after pilocarpine-induced status epilepticus**. (**A**–**D**) Raster plots of the number of spontaneous seizures per day over 42 days of video/EEG recording. Symbols represent the number of seizures observed per day (key in panel (**A**)). (**E**) The average seizure frequency for the same mice: VGAT-Cre, *n* = 3; TRAP2-Cre x CD-1, *n* = 12; Aldh1l1-Cre x CD-1, *n* = 11; and C57BL/6J, *n* = 8. Statistical analysis by one-way parametric ANOVA followed by Dunnett’s multiple comparison test. (**F**) The percentage of seizure-free days for the same mice. Statistical analysis by non-parametric Kruskal–Willis test followed by Dunn’s multiple comparison test. (**G**) The average duration of each spontaneous seizure for each mouse. Statistical analysis by parametric Brown–Forsythe ANOVA followed by Dunnett’s T3 multiple comparison test. (**H**) The persistence of seizure frequency after weeks of recording is strain-dependent. The average seizure frequency per week for each mouse was calculated, then averaged for each strain. The data were fit to equations for either one-phase decay, a straight line, or a horizontal line. The best fits were as follows: TRAP2-Cre, one-phase decay, *p* = 0.001; C57BL/6J, straight line with a slope of −0.25 Sz/wk, *p* = 0.004; VGAT-Cre, horizontal line, *p* = 0.42; and Aldh1l1-Cre, horizontal line, *p* = 0.29. For clarity, only the line is plotted for Aldh1l1-Cre. Abbreviations: **, *p* < 0.01; and ***, *p* < 0.001.

Figure 5 presents the seizure outcomes among mice treated with pilocarpine while on the EEG rig shown in Figure 4. An exception is the results from epileptic VGAT-Cre mice, which were studied before the PK-Pilo model was refined and so mortality was high (60%). We present individual raster plots of each animal from four different strains of mice, where the number of seizures in a day are represented by colored symbols (Figure 5A–D). Days with greater than seven seizures/day are highlighted by a red square. In many cases, these days are followed by no seizures (small clear circle), which is like the clusters observed in rats (Figure 1A). Visual inspection of these rasters indicates that TRAP2-Cre mice showed a high degree of clusters followed by Aldh1l1-Cre mice. Seizure frequency was calculated by dividing the total number of seizures by the days from the first seizure to the end of the EEG recording. Most strains were still seizing at the end of the EEG recording. In general, seizure frequencies ranged between 1 and 2 Sz/d, with lower frequencies observed in C57BL6J mice (Figure 5E). Notably, seizure frequencies varied greatly among TRAP2-Cre mice. The percentage of seizure-free days was calculated by counting the days without seizures and dividing by the days between the first seizure and the end of the recording (Figure 5F). The seizure-free patterns are a mirror image of the seizure frequency plot, with C57BL/6J mice showing the highest percentage of seizure-free days. Surprisingly, we observed a difference in the duration of each seizure, with C57BL/6J having the shortest duration (Figure 5G). As noted in Figure 1F, spontaneous seizures in hybrid kindled mice were transient. To measure this propensity to remission, we calculated the number of seizures observed per week for each mouse, then fitted the data (Figure 5H). As observed in Figure 5B, TRAP2-Cre mice typically started with a high seizure frequency in the first week after pilocarpine, so the results were best fit with a one-phase decay equation. After this initial burst, TRAP2-Cre mice tended to seize regularly about 2/d. In contrast, C57BL/6J mice showed transient epilepsy, with only one mouse, RM912, still seizing at the end of the recording period. Note, mouse RM913 was excluded from this analysis based on the criteria that at least two spontaneous seizures define epilepsy. Seizure frequencies for VGAT-Cre and Aldh1l1-Cre mice were relatively constant around 2 Sz/d over 6 weeks of recording.

## 3. Discussion

### 3.1. Animal Models Suitable for Drug Screening Are an Unmet Need

The goal of these studies was to develop a model of temporal lobe epilepsy where the seizure patterns are amenable to drug screening. Effective seizure control in patients with TLE can be challenging. It is often effectively managed by anti-seizure drugs such as carbamazepine, levetiracetam, and lamotrigine. In addition, there are a myriad of drugs approved as add-on treatments. Despite this large armamentarium, seizures remain poorly controlled in one-third of TLE patients. To address this unmet need, novel compounds need to be screened in animal models that recapitulate the cardinal features of human TLE. The pilocarpine model of TLE is an established model that fulfills these criteria: the seizure onset zone in the hippocampus, EEG patterns, tonic–clonic convulsions behavior, and hippocampal sclerosis [32,33]. Other important features of an epilepsy model amenable to drug screening are as follows: frequent electrographic seizures with a tonic–clonic motor component (>2 Sz/d), regular seizure patterns with no clusters or many consecutive seizure-free days, and maintaining this seizure pattern long enough for protocols that use a cross-over design. An example of the cross-over design being used by the NINDS-funded Epilepsy Therapy Screening Program includes 7 days of control measurements, 5 days of drug treatment, followed by 7 days of washout [22]. Although there is a plethora of studies on the pilocarpine model of TLE, most focus on improving the induction of status epilepticus while reducing mortality, yet few report on how these modifications affect seizure patterns in chronically epileptic animals. The present study describes modifications to the pilocarpine model that improve its usefulness for drug screening.

### 3.2. Pilocarpine Model in Rats

This study begins by documenting seizure patterns in the rat Li/pilocarpine model. We present the results using a variant of the rat pilocarpine model that includes pretreatment with lithium chloride [34], a model that has been used extensively by our collaborators [35,36]. Lithium pretreatment potentiates pilocarpine action in rats, but not mice [37], meaning that 7-fold lower doses of pilocarpine are required to trigger SE, thereby reducing mortality. In our studies, we induced SE with pilocarpine, allowed the animals to recover and develop epilepsy, surgically implanted an EEG recording headset, and then monitored their seizures. Two common seizure patterns that hamper drug screening were observed: clustering and progression. We conclude that drug screening in the rat pilocarpine model is extremely difficult.

### 3.3. Shortcomings of Kindled VGAT-Cre Mice Models

An alternative TLE model we developed used electrically kindled mice of a specific strain, VGAT-Cre [26]. These transgenic mice have Cre recombinase knocked into the vesicular GABA transporter gene [25]. Our studies showed that this knock-in disrupts VGAT expression, resulting in reduced GABAergic tone in hippocampal neurons. Three features of the model are very low mortality, relatively uniform seizure frequencies with little clustering or progression, and very low neuronal death. Seizure frequencies were 1–2 Sz/d. To increase this frequency, we combined IP kainic acid treatment with kindling (hybrid kindling). Although this combination showed higher seizure frequencies, long-term recordings revealed that epilepsy was transient. We deduce that there are unknown secondary processes that convert transient seizures into chronic epilepsy, although we cannot exclude an active process in VGAT-Cre mice that leads to remission. While electrically evoked seizures are useful for studying drug effects [38], we conclude that kindled VGAT-Cre mice are not useful for screening spontaneous seizures.

### 3.4. Developing a Pharmacokinetic Model of Pilocarpine

From our initial attempts of using pilocarpine to induce epilepsy in VGAT-Cre mice, we learned that the doses required to induce SE overlapped with doses that caused death. Importantly, the three mice that survived showed near ideal seizure patterns (R657, R658, and R723, Figure 5A). This glimpse of success drove our efforts to improve the mouse pilocarpine model of TLE. Our priority was to develop a rational pilocarpine dosing scheme that reduced mortality and improved the percentage of mice that develop status epilepticus. To this end, we developed a pilocarpine pharmacokinetic model, relying on the seminal studies of Mazzuferi et al. [30]. To test the model, we chose Swiss CD-1 mice as this outbred strain can survive pilocarpine-induced SE [27]. A cardinal feature that distinguishes the pilocarpine response between CD-1 and VGAT-Cre mice is that CD-1 mice recover from severe running and jumping seizures, while VGAT-Cre mice enter into a tonically constricted state that leads to death by asphyxiation [29]. These findings informed our choice of studying pilocarpine epilepsy in crosses of CD-1 mice with Cre-driver mice. The pharmacokinetic model predicts that brain concentrations of pilocarpine will decrease substantially after 30 min, informing the use of high booster doses [27] over low booster doses [28]. The model is available online (https://doi.org/10.5281/zenodo.16927645).

### 3.5. Pilocarpine Induction on the EEG Rig

A unique feature of our studies is that we studied pilocarpine-induced status epilepticus while recording EEG. This requires a skilled surgeon to implant the EEG recording headset and an EEG facility such as UVA’s Rodent Epilepsy Monitoring Unit [39]. The first key finding was that pilocarpine triggered seizures at lower doses of pilocarpine on the EEG rig compared to off rig (166 vs. 300 mg/kg, respectively. Figure 3A). This finding confirms previous studies using kainic acid to induce seizures [40,41], which implicated immune activation as the probable cause for the increased sensitivity to chemoconvulsants, although other causes include disruption of the blood–brain barrier and stress during EEG recording. Notably, pilocarpine treatment on rig more efficiently induced SE, with less discrete seizures before SE onset, with a higher percentage of mice that developed SE, and with no difference in survival (Figure 3). As observed previously, mice that did not experience SE did not develop SRSs [37]. In these mice, repeated pilocarpine boosters had no effect, suggesting that pilocarpine induced muscarinic receptor desensitization. We speculate that desensitization is a more prominent feature when animals are treated on the rig. In any case, redosing these mice after allowing 3 days of washout successfully induced SE and spontaneous seizure patterns that were similar to those in mice that went into SE on the first attempt. All mice that experienced SE developed spontaneous seizures. There was little or no latency period; in most mice, SRSs started within 24 h of SE induction (Figure 5). This was particularly true with TRAP2-Cre mice, where many had over 30 seizures in the first couple of days after SE. A common misconception in SE studies without EEG recording is that diazepam terminates the seizure. Our studies clearly show this is not the case, as diazepam delivered 1 h after the start of SE did not prevent mice from remaining in electrographic SE for several more hours (Figure 4). An important effect of diazepam is to reduce tonic–clonic seizures, thereby reducing metabolic stress. Chemoconvulsant-induced SE is a major stress on rats and mice, resulting in significant weight loss that can take a week to recover [42,43].

### 3.6. Strain-Dependent Seizure Outcomes of Pilocarpine-Induced SE

We began with VGAT-Cre mice because our earlier studies found that these mice are prone to develop epilepsy [26]. Unfortunately, pilocarpine treatment of homozygous VGAT-Cre mice led to high mortality with a few notable exceptions (Figure 5). Therefore, we switched to Swiss CD-1 mice, which have been shown to withstand pilocarpine-induced SE [27]. In our pilot study, we induced SE off the EEG rig, then performed surgery to implant the headset, and then recorded EEG. The seizure patterns of these CD-1 mice were characterized by low seizure frequencies (1.4 ± 0.3, *n* = 9) and clustering, with a high fraction of seizure-free days (50.6 ± 5.0, *n* = 9; Figure S1). This seizure pattern does not readily support drug screening campaigns; therefore, we examined other strains of mice and performed pilocarpine treatment on the EEG rig. We focused on Cre-driver mice where Cre is expressed from either *Fos* or *Aldh1l1* promoters and crossed these strains with CD-1 to confer resilience to pilocarpine-induced mortality. With the hopes of making the model more accessible, we also studied pilocarpine-induced epilepsy in homozygous C57BL/6J mice. Status could be induced in all three strains with 166 mg/kg pilocarpine. Strain differences were noted during pilocarpine induction, including SE duration, the number of seizures triggered before SE develops, and the percentage of mice that successfully entered SE (Figure 4). Another interesting finding was that pilocarpine could induce SE in many mice without causing tonic–clonic motor seizures, a prominent finding in our studies with C57BL/6J mice (Supplemental Figure S2). As reported previously [30], pilocarpine-induced SE causes hippocampal sclerosis, and in all strains we observed significant loss of NeuN+ cells in CA3, CA1, and the dentate hilus.

### 3.7. Limitations of Study

Drug screening for anti-epileptic drugs using spontaneous seizures as the readout requires seizure patterns with low variability in seizure frequency and few seizure-free days. This is not representative of human seizure patterns, which typically occur in cycles and at lower frequencies [44]. Age is critical for the pilocarpine model, as the treatment of young rodents (<4 weeks old) does not lead to epilepsy, while the treatment of old rodents leads to high mortality [16,42]. Therefore, the pilocarpine model is not representative of late-onset epilepsy in humans. Pilocarpine models focal epilepsies where seizure onset is in the hippocampal and parahippocampal regions [33]; therefore, this model is not useful for screening drugs for generalized epilepsies. The cost of the model is relatively high (animal costs, surgery, EEG); therefore, it will not be used in the “identification” phase of novel targets identified in human multiomics studies [45], but rather its use will be limited to efficacy studies such as those in the “differentiation” phase of the NINDS Epilepsy Therapy Screening Program [38]. Although we studied the strain dependence of seizure outcomes, more studies are required to identify the strain that produces ideal seizure patterns for drug screening. More studies are also required to determine its pharmacological profile using existing anti-epileptic drugs [46].

## 4. Materials and Methods

### 4.1. Animals

All strains of mice were originally obtained from the Jackson Laboratory (Bar Harbor, ME, USA). An early observation that led to our use of Cre-driver mice was that VGAT-Cre mice were more prone to develop epilepsy than common strains such as C57BL/6 [26]. For the present study, we used the following strains of mice: VGAT-Cre (RRID:IMSR_JAX:028862), TRAP2-Cre (Fos^2A-iCreERT2^; RRID:IMSR_JAX:030323), Aldh1l1-Cre/ERT2 (RRID: IMSR_JAX:031008), floxed tdTomato Ai9 reporter mice (RRID: IMSR_JAX:007909); C57BL/6J (RRID: IMSR_JAX:000664), and Jax Swiss Outbred J:ARC(S), herein “CD-1” (RRID: IMSR_JAX:034608). Homozygous VGAT-Cre mice are viable, so we used the F1 generation from breeders via the Jackson Labs. TRAP2-Cre were crossed with Ai9 mice to homozygosity and then maintained as a colony at the University of Virginia. Male TRAP2-Cre x Ai9 mice were crossed with CD-1 females, which are excellent mothers capable of maintaining large litters (>12 pups). Importantly for these studies, CD-1 mice survive pilocarpine-induced status epilepticus [27]. We confirmed this finding with CD-1 mice from the Jackson Laboratory. This was not the case with CD-1 mice from Charles River (Wilmington, MA, USA), which all died after dosing with pilocarpine off rig at either 250 or 140 mg/kg (strain code 022, *n* = 6 per group, 6–7 weeks old). Aldh1l1-Cre mice were engineered using BAC technology and were maintained in the hemizygous state [47]. We bred Aldh1l1-Cre males with CD-1 females, then genotyped for Cre recombinase (JAX protocol 27167). We used male and female mice that were young adults at the time of surgery (7–9 weeks old) and pilocarpine treatment (8–10 weeks old). All mice were given ad libitum access to food and water, normal 12 h light and 12 h dark cycles, and an enriched environment that was cleaned twice a week. Animals were group housed except during EEG recording when they were singly housed in a plexiglass cage during the EEG recording period. They were connected to a flexible tether and commutator (P1 Technologies, Roanoke, VA, USA) to allow for free access food and water. Studies of epilepsy in rats used the Li/pilocarpine model as described previously [48]. Briefly, we used 7–8-week-old adult male Sprague-Dawley obtained from Charles River Laboratories (Wilmington, MA, USA).

### 4.2. Surgery

A detailed description of our surgical procedures and EEG recording headset has been recently published [49]. Briefly, surgeries were performed under isoflurane anesthesia controlled by a SomnoSuite vaporizer (Kent Scientific Corp, Torrington, CT, USA). For surgery, anesthetized rodents were mounted in a Model 940 stereotaxic apparatus equipped with a digital display console (David Kopf Instruments, Tujunga, CA, USA), which was used to guide implantation of the EEG headset. The EEG recording headset included two bipolar Teflon-coated stainless-steel electrodes (791400, A-M Systems, Sequim, WA, USA) and a cerebellar reference electrode. Recording electrodes were placed in each hippocampus at coordinates (from bregma) 3 mm posterior, 3 mm lateral, and 3 mm depth. Electrodes were connected to a six-pin pedestal (P1 Technologies, Roanoke, VA, USA), which was secured to the skull with Optibond eXTRa universal glue (Kerr Dental, Brea, CA, USA) and dental cement (Stoelting, Wood Dale, IL, USA). Animal discomfort was minimized with bupivacaine (topical 0.25%) and ketoprofen (subcutaneous 4 mg/kg). All studies followed protocols approved by the Animal Care and Use Committee of the University of Virginia and ARRIVE guidelines [50]. Video/EEG recording was performed in the University of Virginia Rodent Epilepsy Monitoring Unit located in the Pinn Hall Vivarium.

### 4.3. Pilocarpine Induction

After recovery from surgery (>3 days), mice were tethered to a video/EEG monitoring system (AURA LTM64 using TWin software (v. 4.5.3.23), Grass Technologies, now Natus, Middleton, WI, USA). Their behavior was continuously monitored using infrared video cameras that were linked to the TWin EEG monitoring software, thereby allowing for visual confirmation that electrographic seizure activity coincided with a behavioral seizure. The pilocarpine treatment protocol is summarized in Table 1. To reduce peripheral effects of muscarinic receptor activation by pilocarpine, we administered N-methyl-scopolamine (S8502, Sigma-Aldrich, St. Louis, MO, USA). To prevent possible antagonism of pilocarpine’s central effects [51], we used a low dose (1.75 mg/kg) and a short treatment (20 min) of N-methyl-scopolamine. No difference in chemoconvulsant properties was found between pilocarpine hydrochloride (MedChemExpress, Monmouth Junction, NJ, USA) and pilocarpine nitrate (Spectrum Chemical, New Brunswick, NJ, USA). However, the saline stocks of the nitrate salt precipitated upon freezing, so the hydrochloride salt was preferred. The use of caffeine with pilocarpine [27] was discontinued after the report that caffeine increases the probability of seizure-induced death in epilepsy (SUDEP; [52]). Diazepam (D0899, Sigma-Aldrich, St. Louis, MO, USA) was dissolved in DMSO and then diluted 1:1 with Cremophor EL (C5135, Sigma-Aldrich, St. Louis, MO, USA). For injection, this mixture was diluted 5-fold into saline fortified with 5% dextrose (final ratio of diazepam/Cremophor/saline was 1:1:10). Cremophor was used instead of other solvents such as polyethylene glycol or corn oil [52]. Dextrose was used to provide an energy source during SE. N-methyl-scopolamine and pilocarpine were administered to the peritoneal space, while diazepam was delivered subcutaneously.

The rat pilocarpine induction protocol differed from that used for mice in the following ways: rats were pretreated 24 h in advance with 3 mmol/kg lithium chloride; pilocarpine was only administered once at 50 mg/kg; and the EEG recording headset was implanted 2 weeks after recovery from pilocarpine treatment.

### 4.4. Kindled and Hybrid Kindled Models in VGAT-Cre Mice

Methods for inducing epilepsy in VGAT-Cre mice with electrical stimulation of the hippocampus have been described previously [26,49]. To improve seizure outcomes, we combined electrical kindling with the chemoconvulsant kainic acid (R&D Systems, Minneapolis, MN, USA). The dosing schedule used the repeated low-dose protocol [53], which starts with a 10 mg/kg dose and follows with 5 mg/kg doses every 20 min until seizures are elicited. This was then followed by electrical kindling 6 times every other day using two-second trains consisting of 1 ms pulses, delivered at 50 Hz, with currents set at 1.5× the after-discharge threshold for each mouse (range 20–120 µA). Mice were fully kindled, as defined by five evoked grade 5 seizures with bilateral tonic–clonic motor components [26].

### 4.5. EEG and Behavioral Monitoring

Spontaneous electrographic seizures were defined as evolving spike-wave discharges with greater than 2 Hz frequency that were at least 3 times the baseline amplitude and lasted longer than 10 s. Electrographic seizures were confirmed by the corresponding video, which also allowed for assignment of a behavioral score. Behavioral scoring followed a modified Racine scale as follows: BSS1, normal behavior; BSS2: behavioral arrest, facial twitching, or head bobbing; BSS3, unilateral forelimb clonus; BSS4, bilateral forelimb clonus; BSS5, rearing and loss of motor control; and BSS6, running seizures and involuntary jumping in the cage [54,55]. The end of SE was determined electrographically by both a slowing in frequency to below 1 Hz and a decrease in amplitude of spike-wave discharges, concurrent with losing their steady rhythmicity [55]. The results for seizure frequency, duration, and behavioral score were tabulated in spreadsheets and graphed using Prism software (GraphPad v8.4.3, Boston, MA, USA). Power spectra were generated using LabChart (AD Instruments, Colorado Springs, CO, USA).

### 4.6. Statistical Analysis and Rigor

Statistics were performed using Prism software (GraphPad, Boston, MA, USA). The results were first analyzed for normality to determine whether parametric or nonparametric tests were appropriate. The tests used for each analysis are reported in the figure legend. P values were generated using a two-tailed test. Female and male mice were used in equal proportions and gave similar responses (*p* > 0.5 for SRS freq., seizure-free days, and seizure duration, *n* = 30 male, *n* = 34 female). Historically, female mice were avoided in epilepsy studies due to effects of the hormone cycle on seizures; however, most female mice lose their reproductive cycle after pilocarpine-induced SE [35]. Video/EEG files were manually scored by two observers that were blind to the experimental condition. The number of mice used in each experiment is stated in the figure legends. This number decreases throughout the experiment due to pilocarpine-induced death, SUDEP, and a loss of headsets.

## 5. Conclusions

We developed a pharmacokinetic model of pilocarpine concentration in the brain that proved useful in guiding the induction of SE. We found that pilocarpine induction could be reliably performed with mice while recording their EEG. We compared seizure patterns in chronically epileptic mice, finding that VGAT-Cre mice show promising seizure frequencies, low seizure-free days, and long-lasting epilepsy. We assert that the pharmacokinetic model of pilocarpine and dosing while on an EEG rig provides a useful model for screening new anti-epileptic therapies.

## Figures and Tables

**Table 1:** Pilocarpine protocol.

Variable	Protocol	Reference
Age of mice	8–12 weeks	[16]
2. Scopolamine dose	1.75 mg/kg	[28]
3. Duration of scopolamine pretreatment	20 min	this study
4. Initial pilocarpine dose ^#^	166 mg/kg	this study
5. Subsequent pilocarpine dose	1/2 initial dose	[27]
6. Timing of 2nd pilocarpine dose	30 min	[27]
7. Diazepam dose	10 mg/kg	[20]
8. Timing of diazepam dose	60’ after SE start	[17]
9. Perform SE induction ON or OFF EEG rig	ON rig	this study

^#^ Initial dose must be determined empirically for each mouse strain.

## Data Availability

The data presented in this study are available in this article and upon request. An Excel file containing a modifiable version of the pilocarpine pharmacokinetic model is freely available at https://doi.org/10.5281/zenodo.16927645.

## Supplementary Materials

The following supporting information can be downloaded at: https://www.mdpi.com/article/10.3390/ijms26199540/s1.

## References

[1] Zack M.M., Kobau R. (2017). National and State Estimates of the Numbers of Adults and Children with Active Epilepsy—United States, 2015. MMWR Morb. Mortal. Wkly. Rep..

[2] Boro A., Haut S. (2003). Medical comorbidities in the treatment of epilepsy. Epilepsy Behav..

[3] French J.A., Williamson P.D., Thadani V.M., Darcey T.M., Mattson R.H., Spencer S.S., Spencer D.D. (1993). Characteristics of medial temporal lobe epilepsy: I. Results of history and physical examination. Ann. Neurol..

[4] Klein P., Dingledine R., Aronica E., Bernard C., Blumcke I., Boison D., Brodie M.J., Brooks-Kayal A.R., Engel J., Forcelli P.A. (2018). Commonalities in epileptogenic processes from different acute brain insults: Do they translate?. Epilepsia.

[5] Kwan P., Arzimanoglou A., Berg A.T., Brodie M.J., Allen Hauser W., Mathern G., Moshe S.L., Perucca E., Wiebe S., French J. (2010). Definition of drug resistant epilepsy: Consensus proposal by the ad hoc Task Force of the ILAE Commission on Therapeutic Strategies. Epilepsia.

[6] Wiebe S., Blume W.T., Girvin J.P., Eliasziw M. (2001). A randomized, controlled trial of surgery for temporal-lobe epilepsy. N. Engl. J. Med..

[7] Khoo A., Tisi J., Mannan S., O’Keeffe A.G., Sander J.W., Duncan J.S. (2021). Reasons for not having epilepsy surgery. Epilepsia.

[8] Samanta D., Ostendorf A.P., Willis E., Singh R., Gedela S., Arya R., Scott Perry M. (2021). Underutilization of epilepsy surgery: Part I: A scoping review of barriers. Epilepsy Behav..

[9] Engel J., Wiebe S., French J., Sperling M., Williamson P., Spencer D., Gumnit R., Zahn C., Westbrook E., Enos B. (2003). Practice parameter: Temporal lobe and localized neocortical resections for epilepsy: Report of the Quality Standards Subcommittee of the American Academy of Neurology, in association with the American Epilepsy Society and the American Association of Neurological Surgeons. Neurology.

[10] de Tisi J., Bell G.S., Peacock J.L., McEvoy A.W., Harkness W.F., Sander J.W., Duncan J.S. (2011). The long-term outcome of adult epilepsy surgery, patterns of seizure remission, and relapse: A cohort study. Lancet.

[11] Engel J., McDermott M.P., Wiebe S., Langfitt J.T., Stern J.M., Dewar S., Sperling M.R., Gardiner I., Erba G., Fried I. (2012). Early surgical therapy for drug-resistant temporal lobe epilepsy: A randomized trial. JAMA.

[12] Galanopoulou A.S., Buckmaster P.S., Staley K.J., Moshe S.L., Perucca E., Engel J., Loscher W., Noebels J.L., Pitkanen A., Stables J. (2012). Identification of new epilepsy treatments: Issues in preclinical methodology. Epilepsia.

[13] Kehne J.H., Klein B.D., Raeissi S., Sharma S. (2017). The National Institute of Neurological Disorders and Stroke (NINDS) Epilepsy Therapy Screening Program (ETSP). Neurochem. Res..

[14] Pitkanen A., Buckmaster P.S., Galanopoulou A.S., Moshe S.L. (2017). Models of Seizures and Epilepsy.

[15] Curia G., Longo D., Biagini G., Jones R.S., Avoli M. (2008). The pilocarpine model of temporal lobe epilepsy. J. Neurosci. Methods.

[16] Buckmaster P.S., Haney M.M. (2012). Factors affecting outcomes of pilocarpine treatment in a mouse model of temporal lobe epilepsy. Epilepsy Res..

[17] Chen L.L., Feng H.F., Mao X.X., Ye Q., Zeng L.H. (2013). One hour of pilocarpine-induced status epilepticus is sufficient to develop chronic epilepsy in mice, and is associated with mossy fiber sprouting but not neuronal death. Neurosci. Bull..

[18] Wang L., Liu Y.H., Huang Y.G., Chen L.W. (2008). Time-course of neuronal death in the mouse pilocarpine model of chronic epilepsy using Fluoro-Jade C staining. Brain Res..

[19] Dey D., Eckle V.-S., Vitko I., Sullivan K.A., Lasiecka Z.M., Winckler B., Stornetta R.L., Williamson J.M., Kapur J., Perez-Reyes E. (2014). A potassium leak channel silences hyperactive neurons and ameliorates status epilepticus. Epilepsia.

[20] Lim J.A., Moon J., Kim T.J., Jun J.S., Park B., Byun J.I., Sunwoo J.S., Park K.I., Lee S.T., Jung K.H. (2018). Clustering of spontaneous recurrent seizures separated by long seizure-free periods: An extended video-EEG monitoring study of a pilocarpine mouse model. PLoS ONE.

[21] Goffin K., Nissinen J., Van Laere K., Pitkanen A. (2007). Cyclicity of spontaneous recurrent seizures in pilocarpine model of temporal lobe epilepsy in rat. Exp. Neurol..

[22] West P.J., Thomson K., Billingsley P., Pruess T., Rueda C., Saunders G.W., Smith M.D., Metcalf C.S., Wilcox K.S. (2022). Spontaneous recurrent seizures in an intra-amygdala kainate microinjection model of temporal lobe epilepsy are differentially sensitive to antiseizure drugs. Exp. Neurol..

[23] Sullivan K.A., Vitko I., Blair K., Gaykema R.P., Failor M.J., San Pietro J.M., Dey D., Williamson J.M., Stornetta R.L., Kapur J. (2023). Drug-Inducible Gene Therapy Effectively Reduces Spontaneous Seizures in Kindled Rats but Creates Off-Target Side Effects in Inhibitory Neurons. Int. J. Mol. Sci..

[24] De Deyn P., Marescau B., MacDonald R. (1990). Epilepsy and the GABA-hypothesis a brief review and some examples. Acta Neurol. Belg..

[25] Vong L., Ye C., Yang Z., Choi B., Chua S., Lowell B.B. (2011). Leptin action on GABAergic neurons prevents obesity and reduces inhibitory tone to POMC neurons. Neuron.

[26] Straub J., Gawda A., Ravichandran P., McGrew B., Nylund E., Kang J., Burke C., Vitko I., Scott M., Williamson J. (2020). Characterization of kindled VGAT-Cre mice as a new animal model of temporal lobe epilepsy. Epilepsia.

[27] Vigier A., Partouche N., Michel F.J., Crépel V., Marissal T. (2021). Substantial outcome improvement using a refined pilocarpine mouse model of temporal lobe epilepsy. Neurobiol. Dis..

[28] Arshad M., Naegele J. (2020). Induction of Temporal Lobe Epilepsy in Mice with Pilocarpine. Bio-Protocol.

[29] Wenker I.C., Teran F.A., Wengert E.R., Wagley P.K., Panchal P.S., Blizzard E.A., Saraf P., Wagnon J.L., Goodkin H.P., Meisler M.H. (2021). Postictal Death Is Associated with Tonic Phase Apnea in a Mouse Model of Sudden Unexpected Death in Epilepsy. Ann. Neurol..

[30] Mazzuferi M., Kumar G., Rospo C., Kaminski R.M. (2012). Rapid epileptogenesis in the mouse pilocarpine model: Video-EEG, pharmacokinetic and histopathological characterization. Exp. Neurol..

[31] Guo D., Zeng L., Brody D.L., Wong M. (2013). Rapamycin Attenuates the Development of Posttraumatic Epilepsy in a Mouse Model of Traumatic Brain Injury. PLoS ONE.

[32] Turski W.A., Cavalheiro E.A., Bortolotto Z.A., Mello L.M., Schwarz M., Turski L. (1984). Seizures produced by pilocarpine in mice: A behavioral, electroencephalographic and morphological analysis. Brain Res..

[33] Toyoda I., Bower M.R., Leyva F., Buckmaster P.S. (2013). Early activation of ventral hippocampus and subiculum during spontaneous seizures in a rat model of temporal lobe epilepsy. J. Neurosci..

[34] Jope R.S., Morrisett R.A., Snead O.C. (1986). Characterization of lithium potentiation of pilocarpine-induced status epilepticus in rats. Exp. Neurol..

[35] Joshi S., Sun H., Rajasekaran K., Williamson J., Perez-Reyes E., Kapur J. (2017). A novel therapeutic approach for treatment of catamenial epilepsy. Neurobiol. Dis..

[36] Lawrence C., Martin B.S., Sun C., Williamson J., Kapur J. (2010). Endogenous neurosteroid synthesis modulates seizure frequency. Ann. Neurol..

[37] Muller C.J., Bankstahl M., Groticke I., Loscher W. (2009). Pilocarpine vs. lithium-pilocarpine for induction of status epilepticus in mice: Development of spontaneous seizures, behavioral alterations and neuronal damage. Eur. J. Pharmacol..

[38] Wilcox K.S., West P.J., Metcalf C.S. (2020). The current approach of the Epilepsy Therapy Screening Program contract site for identifying improved therapies for the treatment of pharmacoresistant seizures in epilepsy. Neuropharmacology.

[39] Bertram E.H. (2021). Seizure Monitoring in Rodents. Experimental and Translational Methods to Screen Drugs Effective Against Seizures and Epilepsy.

[40] Tse K., Beamer E., Simpson D., Beynon R.J., Sills G.J., Thippeswamy T. (2021). The Impacts of Surgery and Intracerebral Electrodes in C57BL/6J Mouse Kainate Model of Epileptogenesis: Seizure Threshold, Proteomics, and Cytokine Profiles. Front. Neurol..

[41] Balzekas I., Hernandez J., White J., Koh S. (2016). Confounding effect of EEG implantation surgery: Inadequacy of surgical control in a two hit model of temporal lobe epilepsy. Neurosci. Lett..

[42] Turski L., Ikonomidou C., Turski W.A., Bortolotto Z.A., Cavalheiro E.A. (1989). Review: Cholinergic mechanisms and epileptogenesis. The seizures induced by pilocarpine: A novel experimental model of intractable epilepsy. Synapse.

[43] Gibbs-Shelton S., Benderoth J., Gaykema R.P., Straub J., Okojie K.A., Uweru J.O., Lentferink D.H., Rajbanshi B., Cowan M.N., Patel B. (2023). Microglia play beneficial roles in multiple experimental seizure models. Glia.

[44] Karoly P.J., Rao V.R., Gregg N.M., Worrell G.A., Bernard C., Cook M.J., Baud M.O. (2021). Cycles in epilepsy. Nat. Rev. Neurol..

[45] Chong D., Jones N.C., Schittenhelm R.B., Anderson A., Casillas-Espinosa P.M. (2023). Multi-omics integration and epilepsy: Towards a better understanding of biological mechanisms. Prog. Neurobiol..

[46] Metcalf C.S., Vanegas F., Underwood T., Johnson K., West P.J., Smith M.D., Wilcox K.S. (2022). Screening of prototype antiseizure and anti-inflammatory compounds in the Theiler’s murine encephalomyelitis virus model of epilepsy. Epilepsia Open.

[47] Srinivasan R., Lu T.-Y., Chai H., Xu J., Huang B.S., Golshani P., Coppola G., Khakh B.S. (2016). New Transgenic Mouse Lines for Selectively Targeting Astrocytes and Studying Calcium Signals in Astrocyte Processes In Situ and In Vivo. Neuron.

[48] Burke C.T., Vitko I., Straub J., Nylund E.O., Gawda A., Blair K., Sullivan K.A., Ergun L., Ottolini M., Patel M.K. (2023). EpiPro, a Novel, Synthetic, Activity-Regulated Promoter That Targets Hyperactive Neurons in Epilepsy for Gene Therapy Applications. Int. J. Mol. Sci..

[49] Straub J., Vitko I., Gaykema R.P., Perez-Reyes E. (2021). Preparation and Implantation of Electrodes for Electrically Kindling VGAT-Cre Mice to Generate a Model for Temporal Lobe Epilepsy. J. Vis. Exp..

[50] Kilkenny C., Browne W.J., Cuthill I.C., Emerson M., Altman D.G. (2010). Improving bioscience research reporting: The ARRIVE guidelines for reporting animal research. PLoS Biol..

[51] Pakarinen E.D., Moerschbaecher J.M. (1993). Comparison of the effects of scopolamine and methylscopolamine on the performance of a fixed-ratio discrimination in squirrel monkeys. Pharmacol. Biochem. Behav..

[52] George A.G., Federico A., Gom R.C., Harris S.A., Teskey G.C. (2023). Caffeine exacerbates seizure-induced death via postictal hypoxia. Sci. Rep..

[53] Umpierre A.D., Bennett I.V., Nebeker L.D., Newell T.G., Tian B.B., Thomson K.E., White H.S., White J.A., Wilcox K.S. (2016). Repeated low-dose kainate administration in C57BL/6J mice produces temporal lobe epilepsy pathology but infrequent spontaneous seizures. Exp. Neurol..

[54] Racine R.J. (1972). Modification of seizure activity by electrical stimulation. II. Motor seizure. Electroencephalogr. Clin. Neurophysiol..

[55] Lewczuk E., Joshi S., Williamson J., Penmetsa M., Shan S., Kapur J. (2018). Electroencephalography and behavior patterns during experimental status epilepticus. Epilepsia.

