# Supplementary figures and images for: Spontaneous Seizure Outcomes in Mice Using an Improved Version of the Pilocarpine Model of Temporal Lobe Epilepsy
Source: Int J Mol Sci. 2025 Sep 29;26(19):9540. doi: 10.3390/ijms26199540 (PMC12525131; doi:10.3390/ijms26199540)

Supplemental figures for Gaykema et al.

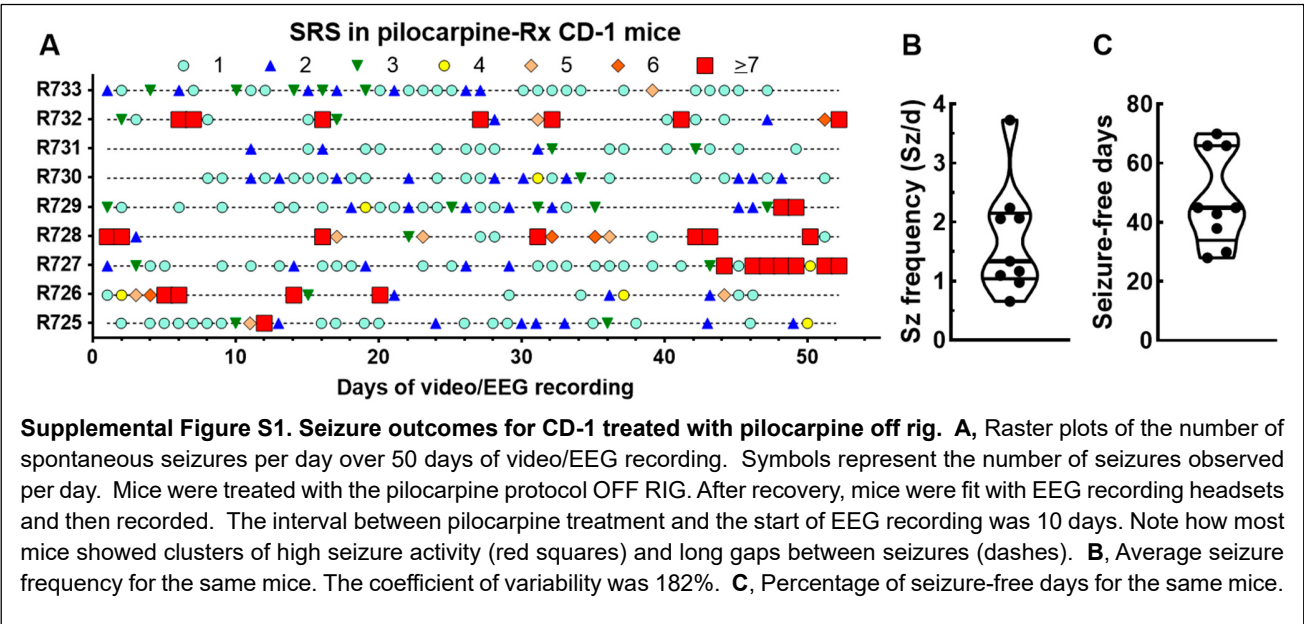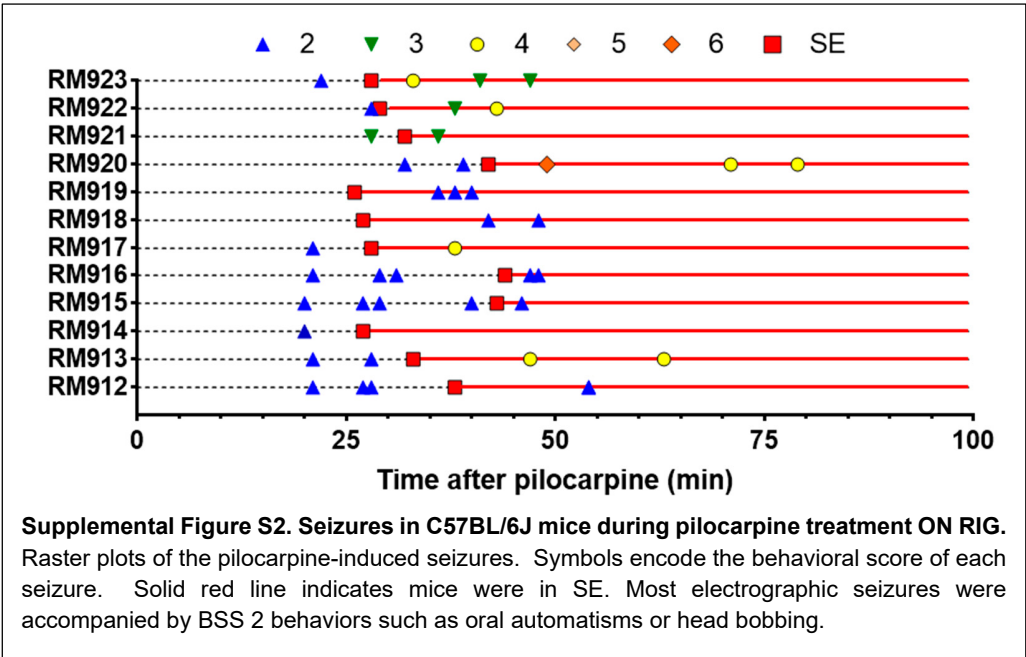

Supplement: Supplementary file 1 [file ijms-26-09540-s001.zip › ijms-3874124-supplementary.pdf]
